# Motivation to Change in the Course of a Pilot Study of a Step-Down Treatment Approach of Inpatient and Anorexia Nervosa-Specific Home Treatment and Its Effects on Treatment Outcome

**DOI:** 10.3389/fpsyt.2021.693103

**Published:** 2021-10-07

**Authors:** Kathrin Sophie Heider, Astrid Dempfle, Sophie Altdorf, Beate Herpertz-Dahlmann, Brigitte Dahmen

**Affiliations:** ^1^Department of Child and Adolescent Psychiatry, Psychosomatics and Psychotherapy, University Hospital, RWTH Aachen University, Aachen, Germany; ^2^Institute of Medical Informatics and Statistics, Kiel University and University Hospital Schleswig-Holstein, Kiel, Germany

**Keywords:** anorexia nervosa, adolescence, treatment setting, home treatment, motivation to change, ANSOCQ

## Abstract

**Introduction:** Anorexia nervosa (AN) is a serious mental disorder that typically manifests in adolescence. Motivation to change is an important predictor for treatment outcome in adolescent AN, even though its development over the often long therapeutic process, with transitions between treatment settings, has not yet been studied. In this pilot study, the course of motivation to change and its effect on treatment outcome were investigated over the course of a step-down treatment approach during a 12-month observation period.

**Methods:** Twenty-one adolescents admitted to inpatient treatment because of AN received multidisciplinary home treatment (HoT) with several weekly visits after short inpatient stabilization. Eating disorder (ED-)specific cognitive [Eating Disorder Inventory 2 (EDI-2) subscales] and physical [% expected body weight (%EBW)] illness severity and motivation to change [Anorexia Nervosa Stages of Change Questionnaire (ANSOCQ)] were assessed at the time of admission, discharge from hospital, at the end of HoT, and at a 12-month follow-up. Changes in motivation over time and its relationship with treatment outcome were investigated.

**Results:** Mean motivation to change improved significantly over the course of treatment from the contemplation stage [2nd stage, mean ANSOCQ sum score 47.26 (SD 17.60)] at admission to the action stage [4th stage, mean ANSOCQ sum score 77.64 (SD 18.97)] at the end of HoT (*p* < 0.001) and remained stable during the follow-up period. At each assessment, higher motivation to change was significantly correlated with lower ED-specific cognitive illness severity (Spearman ρs: −0.53 to −0.77, all *p* < 0.05). Only pretreatment motivation to change significantly predicted ED-specific cognitive illness severity after the first inpatient treatment phase when taking prior illness severity into account.

**Conclusions:** Motivation to change is an important aspect of treatment success in adolescent AN, especially in the early phase of treatment. In addition, home treatment contributed significantly to a higher motivation. Further longitudinal research into how motivation to change in adolescent patients with AN is related to outcome in this often severe and enduring disease and into targeted therapeutic strategies and interventions that reliably enhance the motivation to change in adolescent patients with AN seems promising.

## Introduction

Anorexia nervosa (AN) is a serious mental disorder typically manifesting itself in adolescence with high physical and mental comorbidity (1), and, among all eating disorders (EDs), it has the highest mortality (2). Additionally, at least one in five adolescent patients develops severe and enduring ED (3). Therefore, early and effective treatment is necessary to prevent protracted courses, associated with high individual, familial and societal tolls (4).

Motivation to change is assumed to play a major role in therapeutic engagement (5). Higher motivation to change at admission is related to better treatment outcomes of AN [for reviews see (6, 7)]. In a study by Hillen et al., adolescent patients with a greater motivation to change at admission [investigated with the Anorexia Nervosa Stages of Change Questionnaire (ANSOCQ)] exhibited higher and more rapid weekly weight gain during inpatient treatment until discharge (8). Furthermore, an increase in motivation to change during the first two weeks of inpatient treatment predicted improvements in ED-specific psychopathology after 6 weeks of treatment in adolescents and adults (9). Schlegl et al. identified therapist-rated motivation to change at admission as a positive predictor of clinically significant improvements in ED psychopathology at hospital discharge after an average of 12 weeks in adult inpatients (10).

Motivation to change also seems to improve during treatment: Hillen et al. investigated motivation to change at three time points throughout inpatient treatment and found a significant increase in motivation to change after an average of 15 weeks of inpatient treatment (8). Castro-Fornieles et al. observed a significant increase in motivation to change across an average of 6 weeks of inpatient treatment in adolescent patients with AN (11).

Despite these findings of increased motivation during inpatient treatment, children and adolescents perceive more coercion (8) and often disagree more with hospitalization than adult patients (12). However, there is little research on motivation to change in adolescent patients with AN in treatment settings other than inpatient treatment. Pauli et al. found that higher motivation to change at the beginning of mostly outpatient treatment of adolescents predicted remission of AN after 9 months of treatment (13). Additionally, day-patient treatment in adolescents (14) and in a mixed sample of adolescents and young adults (15) was associated with a significant increase in motivation to change (measured with the ANSOCQ) from admission to discharge. In an uncontrolled case study of adolescent patients with AN who were in day-patient treatment, Simic and colleagues could show a significant increase in self-perceived ability to recover across the treatment program (16). These few but promising findings seem to suggest that treatment settings other than inpatient treatment have a positive impact on motivation to change in adolescent patients with AN, and potentially even more effects.

However, most studies have investigated motivation to change only after a relatively short time of intervention without a follow-up assessment. There are two notable exceptions. First, the study by Castro-Fornieles et al. found that higher motivation to change at discharge from inpatient treatment was a predictor of target weight maintenance 9 months later (11). However, motivation to change was only investigated at admission and discharge, not at follow-up. The second exception is the study by Goldstein et al., who investigated motivation to change throughout a 10-week day-patient treatment and found significant increases in motivation at the 6-month follow-up (14). Nevertheless, these studies are also limited in that they explore motivation to change only during one treatment setting and not among patients across different treatment settings. However, a change in treatment setting is very common, especially in children and adolescents with AN (17). Investigating motivation to change across treatment settings in adolescent patients could lead to interesting insights into the path to recovery to improve both treatments and outcomes for these patients. A rather novel therapeutic concept for adolescent patients with AN is home treatment (HoT). In the current study, HoT was applied as a stepped care treatment setting, comprising a first inpatient stabilization phase followed by an intensive multidisciplinary home-visit program to shorten hospitalizations (18).

In the current study, we aimed to address the aforementioned gaps and to investigate the development of motivation to change in a stepped care approach including the novel method of HoT with a 12-month follow-up. In particular, we were interested in whether our new home treatment method (HoT) would enhance motivation to change. We hypothesized that, first, the motivation to change would increase during inpatient treatment and HoT, second, that motivation to change would be related to ED illness severity and, third, that motivation to change at the beginning of each treatment setting would predict ED illness severity at the end of each setting.

## Materials and Methods

### Study Design and Treatment

The current study was part of a single-center, non-randomized, open-label pilot study that was performed at a tertiary care University clinic department for child and adolescent psychiatry in Germany from May 2017 to December 2019. Our study was approved by the local ethics committee and was undertaken according to the Declaration of Helsinki and Good Clinical Practice (GCP) regulations with independent data management. Written informed consent was obtained from all patients and their parents. The trial was registered in the German Clinical Trials Register (DRKS00013075).

The actual treatment that was offered to adolescent patients with AN can be divided into three phases: (A) an inpatient somatic and mental stabilization phase, followed by (B) the HoT itself, and (C) outpatient treatment as usual (mostly psychotherapy and weight control once a week). The inpatient treatment took place on a specialized ED treatment unit of the department for child and adolescent psychiatry. The treatment setting is multidisciplinary based on ED-specific cognitive-behavioral principles including individual and ED-focused group psychotherapy (psychoeducation, body image therapy, group cognitive behavioral therapy, social skills), weight management and rehabilitation, nutritional therapy with supervised meals and parent training, occupational and art therapy, and physiotherapy. Home treatment (HoT) takes the ED-specific treatment to the individual home of the patient with multiple weekly visits by a multidisciplinary team of medical doctors, psychotherapists, nurses, and nutritional and occupational therapists over three to four months (18). Therapeutic interventions are also based on ED-specific cognitive behavioral principles. During the first two months, each patient is visited three to four times per week, over the third and 4th month one to two times per week, with an additional weekly group therapy session and a mandatory weekly family session [see also (18) for further details]. We assessed the patients and the course of treatment at four time points: a baseline assessment at hospital admission (t_Adm_), a second assessment after inpatient stabilization before starting HoT (t_DisIP_), and at discharge from HoT (t_DisHoT_). A fourth follow-up assessment was scheduled 12 months after admission (t_FU_). The patients' target weight was generally set between the 25th and the 30th age-adapted body mass index (BMI) percentile. More detailed information on the sample, eligibility criteria, assessments, and treatment effects has been reported previously (18). In the current study, we aimed to investigate the development of motivation to change across the treatment and its effects on treatment outcome.

### Participants

Participants were patients with AN or atypical AN according to DSM-5 criteria admitted to the inpatient department due to failure of outpatient treatment or somatic complications caused by starvation. Patients aged between 12 and 18 years, without severe somatic or mental comorbidities, and living with at least one carer within a commute of 60 min of the hospital were eligible to participate. In the second inclusion step, before the beginning of HoT, patients had to be able to eat autonomously (e.g., without nasogastric tube), had exhibited sufficient weight gain (≥1.5 kg in 4 weeks), and were still free of severe mental (e.g., suicidality) or somatic comorbid disorders [for further details see (18)]. 21 of the initially included 22 patients completed HoT and participated in the follow-up assessment. Accordingly, only the data of the 21 patients were included in the current study. Demographic and clinical parameters are displayed in Table 1 (see below).

**Table 1:** Demographic data of the 21 participants.

	**Mean (SD) or *n* (%)**
Age at admission (years)	15.10 (1.16)
Duration of illness (weeks)	50.67 (31.50)
Gender (female)	21 (100%)
AN restrictive subtype	21 (100%)
AN-type	
Typical AN	18 (85.7%)
Atypical AN	3 (14.3%)
Current family situation	
Living with both parents	19 (90.5%)
Living with one parent/patchwork family	2 (9.5%)
Number of inpatient treatments (including current)	
1	18 (85.7%)
2	3 (14.3%)

*SD, standard deviation; n, number; AN, anorexia nervosa*.

### Assessments

#### Motivation to Change

The Anorexia Nervosa Stages of Change Questionnaire (ANSOCQ) is a 20-item self-report questionnaire that assesses the patient's readiness to recover from AN based on the “motivational stages of change model” by Prochaska and DiClemente (19, 20). Every item was answered on a Likert scale ranging from 1 to 5, yielding a sum score between 20 and 100. If sum scores ranging from 20 to 30 are obtained, patients are classified as being in the “Precontemplation” stage, scores from 31 to 50 correspond to the “Contemplation” stage, between 51 and 70 to the “Preparation” stage, between 71 and 90 to the “Action” stage, and scores between 91 and 100 define the “Maintenance” stage. The first stage, “Precontemplation,” represents the unawareness or unwillingness to change, while the second stage, “Contemplation,” refers to recognizing the benefits of change and thinking about change. The “Preparation” stage means that patients intend to change soon and are working on strategies, while the “Action” stage means that patients are already actively working to change and that there is a measurable behavior change. The final stage of change, “Maintenance”, is associated with relapse prevention (19). In our study, we used the German version of the ANSOCQ (13).

#### ED-Specific Cognitive Illness Severity

The Eating Disorder Inventory 2 (EDI-2) is a self-report questionnaire with 91 items mapping onto the following 11 psychological subscales: Body Dissatisfaction, Bulimia, Drive for Thinness, Asceticism, Ineffectiveness, Social Insecurity, Interpersonal Distrust, Perfectionism, Interoceptive Awareness, Impulse Regulation, and Maturity Fears. The psychometric properties of the German version of the EDI-2 have been established by Paul and Thiel (21). The EDI-2 is a validated instrument for AN with good psychometric properties (22). In line with previous research (23–25), we only used the sum score of the three ED-relevant subscales in the analyses: “Drive for Thinness”, “Bulimia”, and “Body Dissatisfaction” as a measure of cognitive ED illness severity. Internal reliability (Cronbach's alpha) of this ED-specific sum score was between 0.63 and 0.73 at the different time points.

ANSOCQ and EDI-2 were administered at baseline (t_Adm_), the end of inpatient treatment (t_DisIP_), the end of home treatment (t_DisHoT_), and 12 months postadmission (t_FU_).

#### ED-Specific Physical Illness Severity

The patient's height and weight (in underwear) were measured at every assessment to calculate the BMI and its age-specific percentile and the percentage of expected body weight (% EBW) [both based on the large German KIGGS study as reference data (26)]. The later was used as the measure of physical ED illness severity.

### Statistics

Statistical analyses were conducted with IBM's Statistical Package for the Social Sciences (SPSS) version 25.0 (IBM Corp., Armonk, USA). The significance level was set to 5%, and the results of the regressions are reported with predictors not explaining any variance (*p* > 0.2) excluded. First, the data were analyzed descriptively.

#### Change in Motivation Over Time

A repeated-measures ANOVA with Bonferroni-adjusted *post hoc* analyses was performed to analyze the change in motivation across the four time points from admission to the one-year follow-up. Exploratively, a descriptive analysis was carried out to detect the numbers of participants whose stage of motivation to change improved, stayed unchanged, or declined between the end of HoT and follow-up.

#### Association Between Motivation to Change and Illness Severity at Each Assessment

To replicate the findings of earlier studies, Spearman correlations were calculated to assess the relationship between motivation to change (ANSOCQ) at admission and other variables at t_Adm_, both measures of illness severity, age, and duration of illness. In addition, the association of motivation to change with both measures of illness severity (EDI-2, %EBW) at each treatment setting change, i.e., at t_DisIP_, t_DisHoT_, and at t_FU_ (after regular outpatient treatment), were also assessed with Spearman correlations.

#### Predictive Association of Motivation to Change for Outcome Illness Severity

To identify whether motivation to change at each treatment stage was associated with illness severity at the following assessment across treatment, three stepwise regressions were performed with cognitive or physical ED illness severity (EDI-2 subscales or %EBW) as the dependent variable, and, in the first step, only the ANSOCQ sum score of the previous assessment and, in the second step, additionally, the ED illness severity (EDI-2 subscales and %EBW) of the previous assessment as well as age as independent variables. For the final model, predictors with *p* > 0.2 in the second step were removed.

## Results

**Figure 1:**
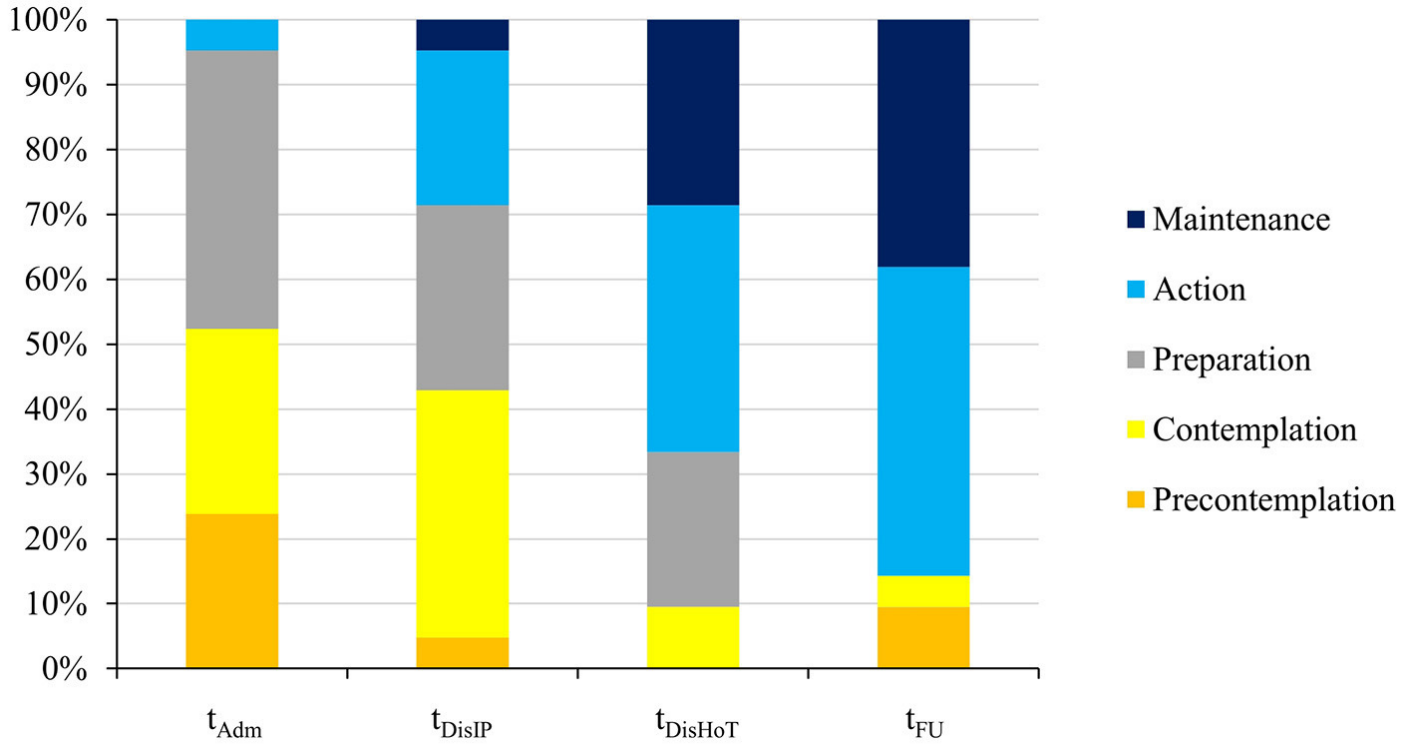
Overview of the distribution of the motivational stages at the different time points according to the ANSOCQ (Anorexia Nervosa Stages of Change Questionnaire). t_Adm_, admission; t_DisIP_, discharge from inpatient treatment; t_DisHOT_, discharge from home treatment; t_FU_, follow-up.

All participants were female and had the restrictive subtype of AN (see Table 1). Inpatient treatment lasted on average 7.5 (SD 1.0) weeks, home treatment 15.5 (SD 1.2) weeks, and outpatient treatment as usual before follow-up 27.7 (SD 3.3) weeks. The main results regarding improvements in ED psychopathology and restoration of body weight during the treatment and follow-up periods have been reported previously (18). The relevant parameters for the current study are shown again for reference in Table 2, together with the development of the motivation to change (ANSOCQ) across assessments. The distribution of the ANSOCQ stages at each assessment is displayed in Figure 1.

**Table 2:** Development of patients' motivation to change, cognitive ED illness severity (EDI-2), BMI and BMI-percentile, and physical illness severity (%EBW) at the beginning of each treatment phase and at the one-year follow-up.

**Indices of illness severity**	**Admission (t_**Adm**_)**	**Start of Home Treatment (t_**DisIP**_)**	**End of Home Treatment (t_**DisHoT**_)**	**1-Year Follow-up (t_**FU**_)**
	***n*** **= 21**			
	***n*** **(%) or mean (SD); Min; Max**			
ANSOCQ sum score	47.26 (17.60); 24.00; 81.50	56.33 (18.31); 24.00; 92.00	77.64 (18.97); 30.00; 99.00	79.38 (21.14); 29.00; 97.00
precontemplation	5 (23.8%)	1 (4.8%)	0	2 (9.5%)
contemplation	6 (28.6%)	8 (38.1%)	2 (9.5%)	1 (4.8%)
preparation	9 (42.9%)	6 (28.6%)	5 (23.8%)	0
action	1 (4.8%)	5 (23.8%)	8 (38.1%)	10 (47.6%)
maintenance	0	1 (4.8%)	6 (28.6%)	8 (38.1%)
Cognitive illness severity (ED-specific EDI-2 sum score)	79.29 (18.85); 31.00; 107.00	72.43 (20.44); 31.00; 107.00	66.43 (19.35); 29.00; 106.00	61.33 (22.39); 25.00; 104.00
BMI	16.32 (1.14); 14.74; 18.61	18.39 (1.02); 16.78; 20.29	19.66 (1.03); 17.57; 21.15	19.72 (1.32); 17.09; 21.91
BMI percentile	3.77 (4.40); 0.01; 14.31	17.63 (10.70); 2.38; 41.06	31.19 (10.17); 12.18; 44.77	28.96 (14.98); 3.87; 62.30
Physical illness severity (%EBW)	78.27 (4.89); 68.98; 86.52	87.83 (4.45); 79.91; 96.70	93.28 (3.76); 86.71; 98.14	92.52 (5.72); 80.77; 105.15

*N, number; SD, standard deviation; Min, minimum; Max, maximum; ANSOCQ, Anorexia Nervosa Stages of Change Questionnaire; ED, eating disorder; BMI, body mass index; %EBW, percentage of expected body weight; EDI-2, eating disorder inventory 2*.

### Change in Motivation Over Time

**Figure 2:**
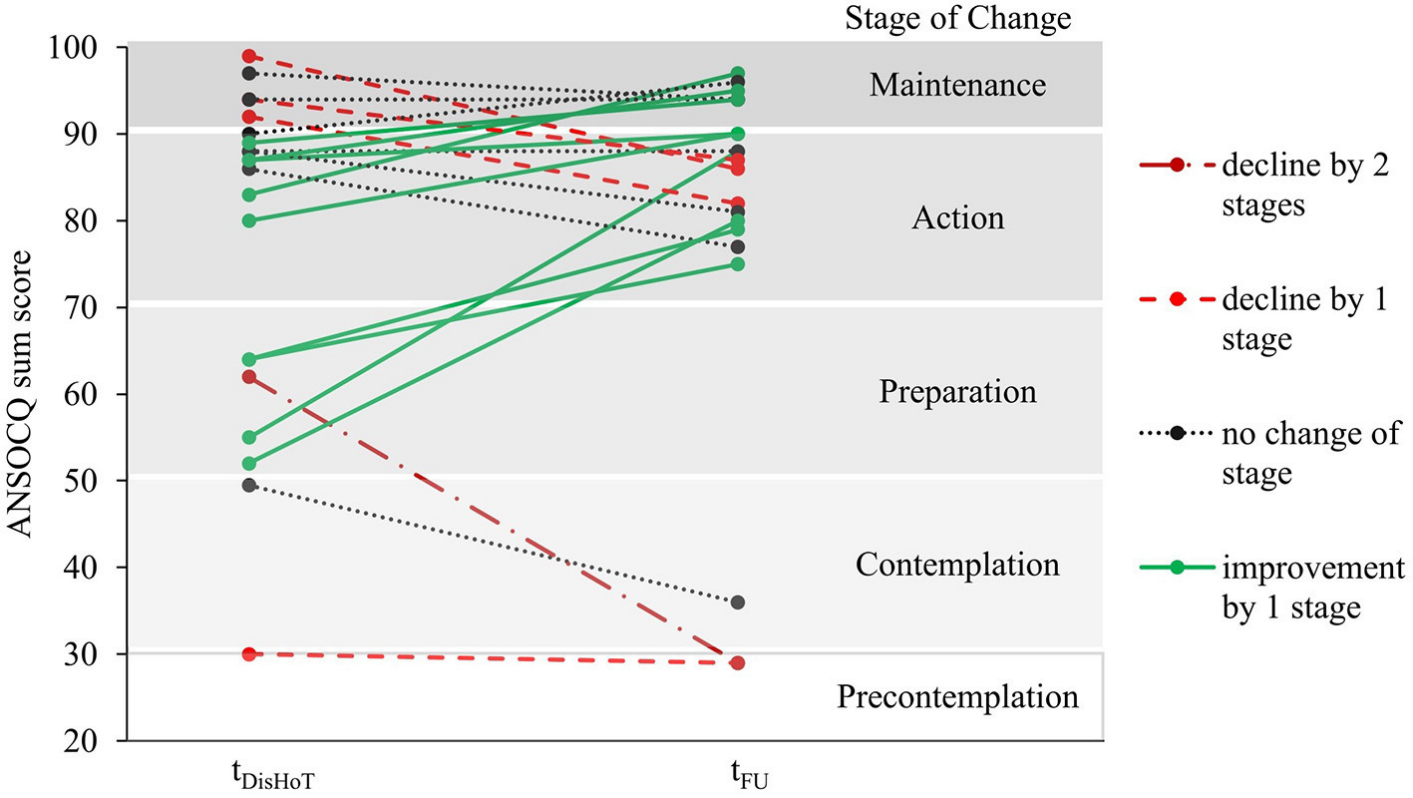
Change in motivational stages of the ANSOCQ of the individual patients between discharge from home treatment and follow-up. ANSOCQ, anorexia nervosa stages of change questionnaire; t_DisHOT_, discharge from home treatment; t_FU_, follow-up.

The patients' motivation to change increased significantly during treatment until the one-year follow-up [*F*_(3, 60)_ = 38.58; *p* < 0.001] from a mean of 47.26 (SD 17.60) at t_Adm_, representing the “Contemplation” stage (2nd stage of the model), to a mean of 79.38 (SD 21.14) at t_FU_, representing the “Action” stage (see Table 2). Bonferroni-adjusted *post hoc* analyses revealed pairwise significant differences between most assessments [e.g., between t_Adm_ and t_DisIP_: −9.07, 95%–CI (−17.29 to −0.85); *p* = 0.025, and between t_DisIP_ and t_DisHoT_: −21.31, 95%–CI (−30.67 to −11.95); *p* < 0.001], except for the period between t_DisHoT_ and t_FU_ [−1.74, 95%–CI (−11.12 to 7.64); *p* = 1.00], when the scores remained stable on average. The largest increase in motivation between consecutive time points occurred during the actual HoT treatment phase (with a mean increase of 21.31 points corresponding to one stage of change). At follow-up, most patients (76.1%) had remained in the same stage of motivation to change or had even increased their motivation: 9 patients (42.9%) improved their stage by 1 until follow-up, 7 patients (33.3%) reported the same stage of motivation to change at follow-up as at the end of the HoT, and 5 patients (23.8%) declined by 1 (4; 19.0%) or 2 (1; 4.8%) stages (see Figure 2).

### Association Between Motivation to Change and Illness Severity at Each Assessment

At t_Adm_, each setting change, and at t_FU_, higher motivation to change was always significantly correlated with less severe cognitive ED illness severity, with consistently strong correlations between −0.53 and −0.77 (see Table 3). The relationship between the physical index of illness severity (%EBW) and motivation to change was generally less pronounced (Spearman ρs: −0.21 to −0.39) and was not significant in our rather small sample. At admission, patients with higher relative body weight tended to have lower motivation, while this trend was reversed at the follow-up (see Table 2). The duration of illness and the participants' age were not significantly associated with the ANSCOQ sum score at admission in this sample.

**Table 3:** Correlation of the ANSOCQ sum scores at all four measuring times with illness severity (ED-specific sum scores of EDI-2 and %EBW) and baseline characteristics.

**Variables and assessment time points**		**t_**Adm**_**	**t_**DisIP**_**	**t_**DisHoT**_**	**t_**FU**_**
ANSOCQ	/EDI-2	−0.611 (0.003)	−0.770 (<0.001)	−0.704 (<0.001)	−0.528 (0.014)
	/%EBW	−0.353 (0.12)	−0.216 (0.35)	−0.214 (0.35)	0.385 (0.09)
	/duration of illness [wks]	0.192 (0.40)	—	—	—
	/age at t_Adm_	0.053 (0.82)	—	—	—

*Spearman correlation coefficient (p-value); ANSOCQ, Anorexia Nervosa Stages of Change Questionnaire; ED, eating disorder; EDI-2, Eating Disorder Inventory 2; %EBW, percentage of expected body weight; t_Adm_, admission; t_DisIP_, discharge from inpatient treatment; t_DisHoT_, discharge from home treatment; t_FU_, follow-up; wks, weeks*.

### Predictive Association of Motivation to Change for Outcome Illness Severity

If only motivation to change was taken into account, its level at the beginning of each setting was significantly associated with cognitive ED illness severity (ED-specific EDI-2 subscales) at the next assessment in an inverse linear manner [see Table 4 (step 1 for all time points)]. Pre-treatment motivation to change at t_Adm_ explained 55.8% of the variance of the cognitive ED illness severity at t_DisIP_. Together with cognitive ED illness severity at hospital admission, it explained 68.9% of the variance of the short-term outcome of cognitive ED illness severity at t_DisIP_. However, this was only the case in the first treatment phase during inpatient treatment. In the later treatment stages, motivation to change did not explain additional variance in cognitive ED illness severity at the consecutive time point if cognitive ED illness severity of the previous assessment was taken into account as well. Neither age nor %EBW of the previous assessment explained variance in cognitive ED illness severity significantly. At none of the assessments did motivation to change predict physical ED illness severity (%EBW) of the next assessment time point.

**Table 4:** Results of the stepwise regressions of the motivation to change (ANSOCQ sum scores) with cognitive ED illness severity (ED-specific sum score of the EDI-2) over consecutive assessments.

**Predictors and assessment time point**	***R*^**2**^-change**	**B**	**SE**	***P***
**Inpatient treatment. Association between motivation to change and cognitive ED illness severity**.				
Step 1	0.56			<0.001
Motivation t_Adm_		−0.87	0.18	<0.001
Step 2	0.14			0.109
Motivation t_Adm_		−0.51	0.22	0.036
Cognitive ED illness severity t_Adm_		0.49	0.21	0.035
%EBW t_Adm_		0.26	0.61	0.910
age		0.76	2.63	0.852
Step 3^1^	0.69			0.018
Motivation t_Adm_		−0.52	0.20	0.018
Cognitive ED illness severity t_Adm_		0.51	0.19	0.013
Total R^2^ (adj.)	0.66			
**Home treatment. Association between motivation to change and cognitive ED illness severity**.				
Step 1	0.41			0.002
Motivation t_DisIP_		−0.67	0.19	0.002
Step 2	0.27			0.020
Motivation t_DisIP_		−0.12	0.25	0.627
Cognitive ED illness severity t_DisIP_		0.60	0.24	0.021
%EBW t_DisIP_		0.63	0.66	0.357
age		3.23	2.48	0.211
Step 3^*a*^	0.63			<0.001
Cognitive ED illness severity t_DisIP_		0.75	0.13	<0.001
Total R^2^ (adj.)	0.61			
**Outpatient treatment. Association between motivation to change and cognitive ED illness severity**.				
Step 1	0.21			0.036
Motivation t_DisHoT_		−0.54	0.24	0.036
Step 2	0.14			0.377
Motivation t_DisHoT_		−0.25	0.33	0.430
Cognitive ED illness severity t_DisHoT_		0.47	0.39	0.077
%EBW t_DisHoT_		0.51	1.53	0.742
age		−0.88	4.22	0.837
Step 3^*a*^	0.32			0.008
Cognitive ED illness severity t_DisHoT_		0.65	0.22	0.008
Total *R^2^* (adj.)	0.28			

*ANSOCQ, Anorexia Nervosa Stages of Change Questionnaire; ED, eating disorder; EDI-2, Eating Disorder Inventory 2; t_Adm_, admission; %EBW, percentage of expected body weight; t_DisIP_, discharge from inpatient treatment; t_DisHoT_, discharge from home treatment*;

a *final model, predictors with p > 0.2 (motivation in the 2nd step) were removed*.

## Discussion

In our prospective pilot study, motivation to change increased significantly during all treatment phases of the step-down approach of brief somatic inpatient stabilization followed by the novel treatment of AN-specific HoT and remained stable until follow-up. At each assessment stage, higher motivation to change was significantly related to cognitive ED illness severity in a negative inverse manner. Only in the first phase of treatment did motivation to change prove to be a significant additional predictor of cognitive ED illness severity at the end of the inpatient treatment phase. To the best of our knowledge, this is the first study to investigate motivation to change comprehensively in different treatment settings over a 12-month period. According to our results, the new method of home-based treatment showed the largest increase in motivation to change, which assures us that it is a valuable strategy to enhance the compliance of adolescent patients with AN.

In our sample, motivation to change increased significantly across treatment. This finding is in line with previous studies of adolescent and adult patients with AN in inpatient, outpatient, and day-patient treatments (9, 14–16, 27). Hillen et al. reported a significant increase in motivation to change over an average inpatient treatment period of 15.1 weeks (8). In Castro-Fornieles et al.'s study, the patients reported a significantly improved motivation to change after approximately 4 weeks of inpatient treatment (11). Goldstein et al. reported significant increases in motivation to change after approximately 10 weeks of day-patient treatment and at a 6-month follow-up (14). Nonetheless, our study extends the previous findings by showing consistent significant increases across several treatment setting changes. Between discharge from HoT and the follow-up, three in four patients remained in their stage of motivation to change or improved, while only one in four patients declined in their stage. Our sample size did not allow further subsample analyses, but, if replicated, future studies with larger samples could investigate a possible clinical implication of change in motivation after discharge.

A finding that has not yet been previously reported is the great increase in motivation to change in our patient sample from hospital admission to the start of HoT and further until the end of HoT by an average of 30 points, corresponding to ~1.5 stages of change. The mean score at admission of our sample was even slightly lower than those in other studies (8, 11, 14). The mean ANSOCQ sum scores at discharge (77.64) and follow-up (79.38) in our study were higher than the scores of all other studies, which reported mean values. Only in the studies of Hillen et al. (at discharge) and Goldstein et al. (at the 6-month follow-up) did adolescent patients with AN achieve mean values higher than 70 points, indicating the “Action” stage of change (8, 14). This may emphasize that motivation to change increased especially during our new treatment program: home-based treatment might motivate the patients more than treatment as usual because it facilitates the return home and helps to maintain the adolescents' social lives. It has been shown previously that friendships and peer support are positively associated with motivation to change in patients with AN (28), which might underlie this HoT-specific increase. Also, the patients and families actively opted for HoT as their treatment setting of choice; active involvement in treatment organization probably enhances motivation to change in patients with AN (5). In addition, motivation to change might have changed because of the long-term psychotherapeutic treatment at home of approximately 22 weeks.

Higher motivation to change at admission was associated with less severe ED-specific symptoms at admission. This finding is in line with prior studies (8, 13): Pauli et al. found significant negative correlations between the ANSOCQ and the EDI-2 “Drive for Thinness” scale at admission to outpatient treatment in adolescents with AN (13). In addition to similar findings between motivation and ED illness severity at admission, Hillen et al. also reported significant associations of higher motivation to change with lower %EBW at admission and longer duration of illness (8). Patients with a longer duration of illness and severe underweight might have experienced more somatic and psychological strain before admission, leading to higher motivation to change in comparison with patients with better somatic health. In our sample, at admission, lower %EBW in the patients also tended to be associated with higher motivation in the patients, but the relationship was not significant in our rather small sample. There was also no association between duration of illness and motivation to change.

The difference in sample size might partially explain the difference in these findings. Moreover, the duration of illness had a larger range (3–40 months) in Hillen et al.'s cohort than in our sample (1–27 months). Interestingly, the trend of the association of lower %EBW with higher motivation at admission was reversed in our sample at follow-up, with higher motivation being at trend-level significance associated with higher %EBW, suggesting an association between higher motivation to change at follow-up and better maintenance of %EBW.

Similar to previous findings, higher motivation to change at admission was predictive lower cognitive ED illness severity after inpatient treatment, cognitive ED illness severity at admission into account [e.g., (9)]. During the later stages of our step-down treatment approach, the relationship between motivation to change at the beginning cognitive ED illness severity at the end of each treatment phase, adjusted for prior cognitive ED illness severity, was not significant. This corresponds to a result by Ackard et al., who did not find that stage of change predicted ED-specific illness severity at 3-, 6-, and 12 months follow-up although they used a different instrument than the ANSOCQ (29). A potential explanation might be that high motivation to change facilitates early improvement independent of illness severity, while for long-term recovery, individual illness severity might be a more relevant predictor. However, considering the observational nature of our study and the strong correlations between motivation to change and cognitive ED-specific illness severity at each assessment, it is not completely clear which factors were primarily responsible for the improvement in illness severity during the later treatment phases.

Motivation to change of the previous assessment did not predict weight as measured by %EBW at any stage of the treatment process in our study, which contrasts with other studies which reported an association [e.g., (11)]. This difference might be explained by large differences in initial weights at admission because of the inclusion of patients with atypical AN. Moreover, a steady weight gain during inpatient treatment was required to be transferred to HoT, and all patients had agreed to achieve their target weight during HoT.

Some limitations must be considered when interpreting the results. As mentioned already, our sample size of 21 participants was small because it was a pilot and observational study to investigate the effects of a new treatment method. To confirm our results, especially whether HoT contributes more to motivation to change in comparison with other intensive treatment settings, randomized controlled trials seem to be necessary. In addition, although the ANSOCQ is a validated and suitable measure to assess motivation to change in the context of AN (27), other assessment instruments were used in previous studies, which might hinder the comparability of studies.

The results of our pilot study suggest several interesting questions for future studies. Considering the large body of evidence for an association between motivation to change and treatment outcome in patients with AN, we suggest that motivation to change should be investigated in longer-term follow-up studies because of its potential to detect a risk of relapse and enable timely intervention (30). Additionally, it might be helpful to investigate the efficacy of (novel) treatment concepts or settings for patients with AN regarding their ability to improve the motivation to change, especially in the early phases of treatment.

To conclude, motivation to change in an adolescent patient sample with AN increased significantly during a step-down treatment approach with changes in treatment settings, including short inpatient treatment and a novel intensive HoT program. Our results suggest that after discharge from this intensive treatment program (HoT), the motivation to change remained stable and was associated with reduced cognitive ED illness severity. Further research into targeted therapeutic strategies and interventions that reliably enhance the motivation to change in patients with AN seems promising to improve the outcome of this often severe, enduring, and disabling disease.

## Data Availability Statement

The raw data supporting the conclusions of this article will be made available by the authors, without undue reservation.

## Ethics Statement

The studies involving human participants were reviewed and approved by Ethics Committee of the medical faculty, RWTH University Hospital Aachen, Germany. Written informed consent to participate in this study was provided by the participants' legal guardian/next of kin.

## Author Contributions

BH-D, AD, and BD designed the study. SA and BD enrolled patients into the study and contributed to data collection. KH assisted with data collection. KH did the statistical analysis and AD and BD supervised it. BD, BH-D, AD, and KH interpreted the data. KH wrote the original draft of the paper and BD revised it. BH-D, AD, SA, KH, and BD critically reviewed and corrected the paper. All authors contributed to the article and approved the submitted version.

## Funding

Funding was provided by the Ministry of Labor, Health, and Social Policies of the State of North-Rhine-Westphalia, Germany. The funding source had no role in the design of the study, its execution, data analyses, and interpretation, or decision to submit results.

## Conflict of Interest

BH-D received a speaker's fee from Ferring for a disease state presentation. The remaining authors declare that the research was conducted in the absence of any commercial or financial relationships that could be construed as a potential conflict of interest.

## Publisher's Note

All claims expressed in this article are solely those of the authors and do not necessarily represent those of their affiliated organizations, or those of the publisher, the editors and the reviewers. Any product that may be evaluated in this article, or claim that may be made by its manufacturer, is not guaranteed or endorsed by the publisher.

## Abbreviations

AN: anorexia nervosa
ANSOCQ: Anorexia Nervosa Stages of Change Questionnaire
BMI: body mass index
ED: eating disorder
EDI-2: Eating Disorder Inventory 2
HoT: home treatment
t_Adm_: time point of admission
t_DisIP_: time point of discharge from inpatient treatment/time point of start of HoT
t_DisHoT_: time point of discharge from HoT
t_FU_: time point of follow-up, 12 months after admission
%EBW: percentage of expected body weight.
